# Is there a relationship between wound infections and laceration closure times?

**DOI:** 10.1186/1865-1380-5-32

**Published:** 2012-07-26

**Authors:** Muhammad Waseem, Viraj Lakdawala, Rohit Patel, Ramnath Kapoor, Mark Leber, Xuming Sun

**Affiliations:** 1 Department of Emergency Medicine, Lincoln Medical & Mental Health Center, 234 East 149th Street, Bronx, NY, 10451, USA; 2 Department of Public health, Weill Medical College of Cornell University, 402 E. 67th Street, New York, NY 10065, USA

## Abstract

**Background:**

Lacerations account for a large number of ED visits. Is there a “golden period” beyond which lacerations should not be repaired primarily? What type of relationship exists between time of repair and wound infection rates? Is it linear or exponential? Currently, the influence of laceration age on the risk of infection in simple lacerations repaired is not clearly defined. We conducted this study to determine the influence of time of primary wound closure on the infection rate.

**Methods:**

This is a prospective observational study of patients who presented to the Emergency Department (ED) with a laceration requiring closure from April 2009 to November 2010. The wound closure time was defined as the time interval from when the patient reported laceration occurred until the time of the start of the wound repair procedure. Univariate analysis was performed to determine the factors predictive of infection. A non-parametric Wilcoxon rank-sum test was performed to compare the median differences of time of laceration repair. Chi-square (Fisher's exact) tests were performed to test for infection differences with regard to gender, race, location of laceration, mechanism of injury, co-morbidities, type of anesthesia and type of suture material used.

**Results:**

Over the study period, 297 participants met the inclusion criteria and were followed. Of the included participants, 224 (75.4%) were male and 73 (24.6%) were female. Ten patients (3.4%) developed a wound infection. Of these infections, five occurred on hands, four on extremities (not hands) and one on the face. One of these patients was African American, seven were Hispanic and two were Caucasian (*p* = 0.0005). Median wound closure time in the infection group was 867 min and in the non-infection group 330 min (*p* = 0.03).

**Conclusions:**

Without controlling various confounding factors, the median wound closure time for the lacerations in the wound infection group was statistically significantly longer than in the non-infection group.

## Background

Lacerations account for a significant number of ED visits. In 2002, more than 11 million patients were treated in EDs in the US for lacerations, representing up to 8% of all ED presentations [1,2]. The optimal time interval from injury to laceration repair has not been clearly defined [3]. Although it is generally accepted that the longer the time interval from injury to closure of a laceration is,the higher the incidence of wound infection, there is no clear standard for the "cutoff time" after which primary wound closure should not be performed. The authors seek to determine whether there is an optimal time from injury to laceration repair after which primary wound closure should not be performed because of an unacceptable risk for wound infection. There is currently uncertainty in the existing literature about the “golden period” for laceration repair, and the optimal length of the wound closure time has not yet been adequately defined. Our secondary objective was to determine the possible influence of other factors on the rate of wound infection.

## Methods

### Study design

We performed a prospective observational study of patients with traumatic lacerations to determine the optimum or maximum time for laceration repair.

### Study setting and population

This study was conducted in the Emergency Department of Lincoln Medical & Mental Health Center in Bronx, New York, a level-1 trauma center with an annual adult census of over 120,000 patients. This study included patients who presented to the ED for laceration repair. Patients presented with lacerations between April 2009 and November 2010, and were prospectively enrolled. A trained research assistant or Emergency Medicine resident identified and enrolled potential study participants between the hours of 8 a.m.and 8 p.m., 5 days per week. Between 8 p.m.and 8 a.m., and during weekends, patients were identified and enrolled by physicians (attending or resident) caring for the patient.

The inclusion criteria included all patients who were at least 18 years of age, who presented to the ED with a laceration due to trauma, provided consent, agreed to return for a follow-up visit and provided a reliable history for the time of laceration occurrence. Additionally, they were included if the laceration appeared clean at the time of presentation, the laceration was simple (without associated injuries such as tendon injury, fracture and tissue loss, and not requiring deep sutures). The lacerations were repaired by either a board-certified or board-eligible Emergency Medicine attending physician or Emergency Medicine resident. Exclusion criteria included the following: lacerations showing signs of infection at presentation; animal or human bites; complex lacerations; those repaired by a surgical sub-specialist; oral, mucosal or lip lacerations; eyelid lacerations; puncture wounds; grossly contaminated lacerations; lacerations in patients who received antibiotics at the time of repair; inability to return for the follow-up visit at the study center; refusal to participate in this study. In addition, lacerations repaired by other alternative methods, such as tissue adhesives or adhesive tapes, were also excluded.

### Data collection

After obtaining informed consent, the following information was recorded: medical record number, age, gender, race, laceration location (scalp, face, trunk or extremities, including hands and feet), description of the laceration (length and shape), time of injury, mechanism of injury (blunt versus sharp) and suspicion of foreign body. Time of consent was used to estimate the time of laceration repair. The wound closure time was defined as the interval of time between the time of injury and the time the procedure to repair the laceration was started. From the procedure note, other relevant information was also obtained: the method of wound closure used (simple interrupted sutures, mattress sutures or subcutaneous sutures), the type of suture material used, and whether or not epinephrine was used.

At this center, the method and technique of laceration repair are largely standardized. Wounds are prepared with Betadine swabs over the skin only, anesthetized locally with 1% lidocaine with or without epinephrine (brand, manufacturer of lidocaine (Hospira Inc, Lake Forest, IL)), irrigated copiously with either normal saline or sterile water, repaired with nylon sutures, deep Vicryl absorbable subcutaneous sutures if needed and dressed with sterile gauze after bacitracin application. Repair was performed under sterile conditions. All patients received standardized wound care discharge instructions in English or Spanish.

Patients were followed up at our Wound Care Clinic, which is staffed by our Emergency Medicine Faculty and Residents. Time to follow-up appointment is provided based on the location of laceration unless the presence of other factors warrants an earlier appointment. At our institution, the following timelines are used for scheduling a follow-up appointment:3 to 5 days for facial lacerations, 7-10 days for trunk and extremity lacerations, and 10-14 days follow-up for joint lacerations. At the follow-up visit, the repaired wounds were assessed and classified as either non-infected or infected. For study purposes, infection was defined as the presence of an abscess, purulent drainage or cellulitis more than 1 cm beyond the wound edges requiring antibiotic prescription [4]. The study was approved by the Institutional Review Board at Lincoln Medical & Mental Health Center, Bronx, New York.

### Sample size

Sample size was determined by statistics software PASS 2008 (Kaysville, Utah). With a power of 80% and a significance level of 0.05, 233 patients were required to determine an increase of infection rate from 4% to 8%. Considering a 10% dropout rate, 259 subjects were needed.

### Data analysis

Various analyses were performed to determine whether the time interval of laceration repair would be related to infections after controlling for laceration site, length of laceration, age, gender and race. The small sample size of the study limits the applicability of the tests based on the assumption that the sampling distribution is normal. There is no way to test this assumption as the study sample size is small. Since data were not normally distributed, non-parametric Wilcoxon rank-sum tests were performed to compare the median differences of time of laceration in the group with infection to the group with no infection. Similarly, the Wilcoxon rank-sum test was applied to compare median ages and median length of laceration between both groups.

For the comparison of the time interval of laceration repair by the binary outcome of presence or absence of infection controlled by variables of gender, race and site, chi-square tests or logistic regression is commonly used in this type of study. However, because of the small sample size and the presence of zero cells, regular logistic regression is invalid and not estimable. No logistic regression was performed. Chi-square tables were used with Fisher's exact test because of the small expected values. All *p*-values were twosided with statistical significance evaluated at the 0.05 alpha levels. All analyses were performed in SAS version 9.2 (SAS Institute, Inc., Cary, NC).

## Results

**Figure 1:**
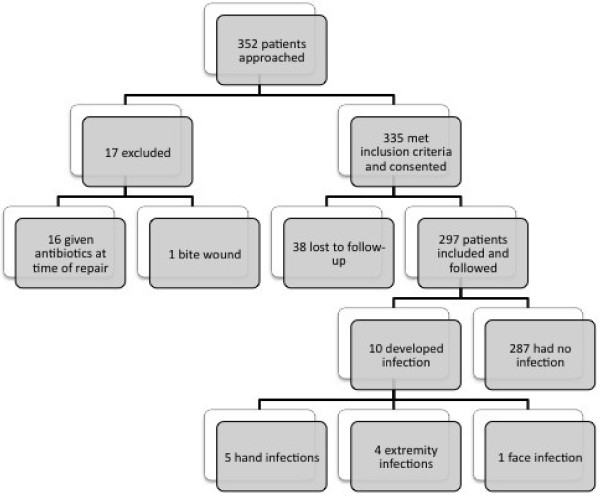
Study Procedures and Enrollment.

Two hundred ninety-seven patients with uncomplicated lacerations met inclusion criteria and were followed successfully (Figure 1,flow chart). Patients’ characteristics comparing the infection group with non-infection are described in Table 1. Median age of both infection and non-infection patients was 34 years. Regarding race, in the infection group, one patient was African American, seven were Hispanic and two were Caucasian. In the non-infection group, 94 were African American, 188 were Hispanic and 5 were Caucasian. Differences between the races with or without infection were statistically significant (Fisher’s exact test, *p* = 0.0005). Of the study subjects, 224 (75.4%) were male and 73 (24.6%) were female. In the infection group, 8 (80%) were male and 2 (20%) were female compared to 216 (75.3%) male and 71 (24.7%) female in the non-infection group (Fisher’s exact test, *p* = 0.73).

**Table 1:** Patients’ characteristics according to infection or non-infection in the study

**Characters**		**Number of infection**		
		**Yes (%)**	**No (%)**	*P*-values*
Age	[median (mean ± SD)]	34.0 (34.6 ± 9.6)	34.0 (38.0 ± 15.1)	0.73**
Race				
	African American	1 (10%)	94 (32.8%)	0.0005
	Hispanic	7 (70%)	188 (65.5%)	
	White	2 (20%)	5 (1.7%)	
Gender				
	Male	8 (80%)	216 (75.3%)	0.73
	Female	2 (20%)	71 (24.7%)	
Location				
	Scalp	0 (0%)	26 (9.1%)	0.33
	Face	1 (10%)	83 (28.9%)	
	Trunk	0 (0%)	6 (2.1%)	
	Extremity	4 (40%)	56 (19.5%)	
	Hand	5 (50%)	116 (40.4%)	
Mechanism				
	Sharp	9 (90%)	242 (84.3%)	0.62
	Crush	1 (10%)	45 (15.7%)	
Co-morbidities				
	None	7 (70%)	257 (89.5%)	0.15
	Diabetes	1 (10%)	9 (3.2%)	
	Immunocompromised	0 (0%)	5 (1.7%)	
	Other	2 (20%)	16 (5.6%)	
Anesthesia				
	Lidocaine 1%	7 (70%)	239 (83.3%)	0.54
	Lidocaine with epinephrine	1 (10%)	16 (5.6%)	
	Digital block	2 (20%)	32 (11.1%)	
Suture				
	0 = Nonabsorbable only	10 (100%)	256 (89.2%)	0.54
	1 = Absorbable	0 (0%)	7 (2.4%)	
	2 = Staples	0 (0%)	24 (8.4%)	

*Chi-square or Fisher’sexact test.

** Wilcoxon-Mann-Whitney test.

Of the ten patients (3.4%) who developed infection after laceration repair, five occurred in the hand, four in the extremities (not including the hands) and one on the face. In the non-infection group, 26 (9.1%) were on the scalp, 83 (28.9%) were on the face, 6 (2.1%) were on the trunk, 56 (19.5%) involved extremities (excluding hands or feet) and 116 (40.4%) were on the hands. In the infection group, nine patients (90%) had lacerations due to a sharp object and one patient (10%) had a laceration due to a crush injury. In the other group, 242 (84.3%) had the mechanism of injury secondary to a sharp object and 45 (15.7%) had crush injury.

Regarding co-morbidities in the infection group, three of the ten patients had diabetes or other medical conditions. Seven (70%) patients had no comorbidity. In the non-infection group, 9 (3.2%) had diabetes, 5 (1.7%) were immunocompromised and 16 (5.6%) had other medical conditions such as asthma. Two hundred fifty-seven (89.5%) had no previous comorbid conditions. Using Fisher’s exact test, a comparison of both groups was performed; there was no statistical significance of co-morbidities between the infection and non-infection group (*p* = 0.15).

Regarding the types of anesthesia used, seven (70%) of the ten patients in the infection group received lidocaine 1%, one (10%) was given lidocaine with epinephrine and two (20%) were treated with a digital block. In the other group, 239 (83.3%) received 1% lidocaine, 16 (5.6%) were given lidocaine with epinephrine and 32 (11.1%) were treated with digital block. Fisher’s exact test showed that there was no statistical significance of types of anesthesia used between infection and non-infection group (*p* = 0.55).

All ten patients in the infection group had their lacerations repaired with non-absorbable sutures. In the non-infection group, lacerations were repaired with non-absorbable sutures in 256 (89.2%) patients and absorbable sutures in 7 (2.4%), and staples were applied in 24 (8.4%) patients. Fisher’s exact test showed that there was no statistical significance of types of sutures used between the infection and non-infection group (*p* = 0.54).

**Figure 2:**
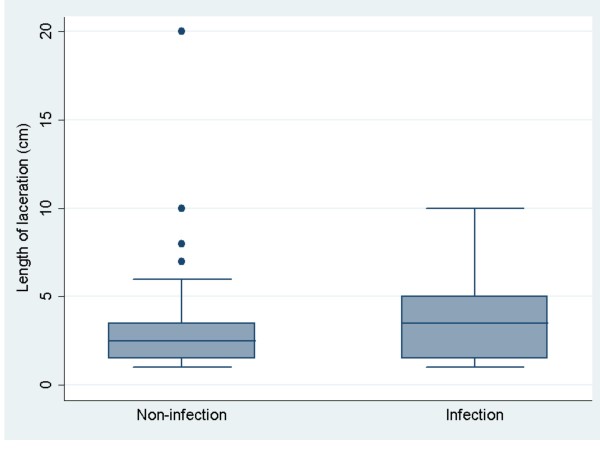
Shows box plots distribution of length of laceration between the infection and non-infection group.

For the continuous variable, median wound length in the infection group was 3.5 cm and 2.5 cm in the non-infection group. Figure 2 shows box plots illustrating the distribution of length of laceration in the infection and non-infection groups. The median length of laceration in the non-infection group was 2.5 cm compared to 3.5 cm in the infection group (Wilcoxon rank-sum test, *p* = 0.17). Twenty-five percent of the total non-infection patients had laceration lengths of less than 1.5 cm, and 75 percent of the total non-infection patients had lengths less than 3.5 cm. The 25th and 75th percentiles for laceration length in the infection group were 1.5 cm and 5.0 cm, respectively.

**Figure 3:**
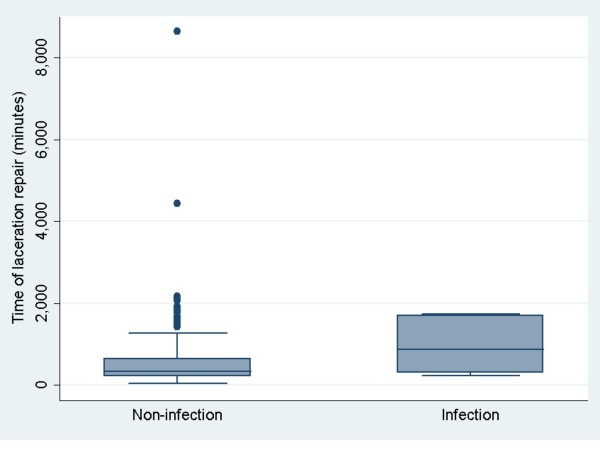
Shows box plots distribution of time of laceration between the infection and non-infection group.

**Figure 4:**
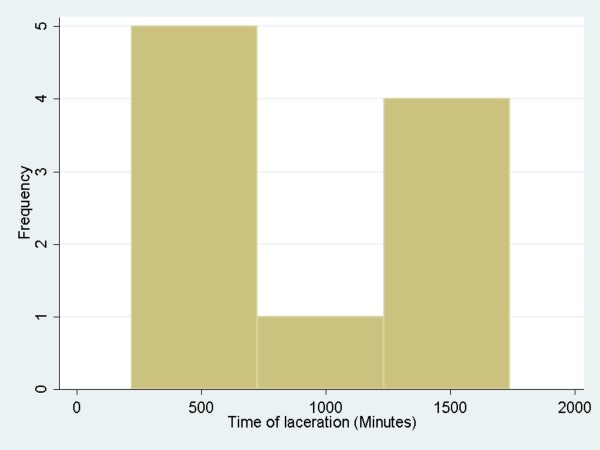
Shows time of laceration repair in patients with wound infection.

**Figure 5:**
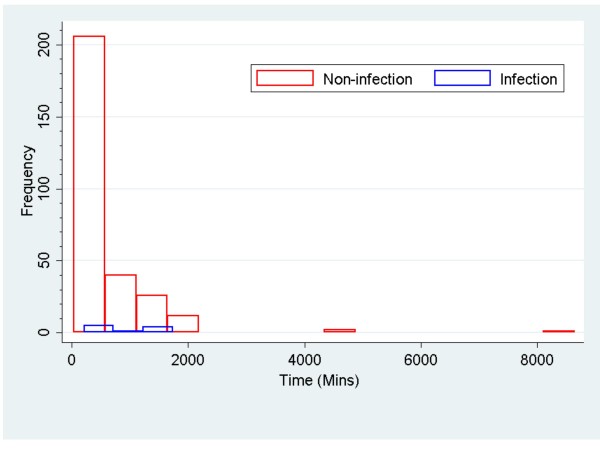
Shows combined histograms comparing distribution of frequencies of infection and non-infection group.

Median wound closure time for the lacerations in the infection group was 867 min and in the non-infection group was 330 min (Table 2). Figure 3 shows box plot distributions of time of laceration repair for the infection and non-infection groups. Median for time of laceration repair in the non-infection group was 330 mins compared to 867 min in the infection group (Wilcoxon rank-sum test, *p* = 0.03). Time of repair less than 215 min comprised 25 percent of the total non-infection patients and less than 645 min constituted 75 percent of total non-infection patients. The 25th and 75th percentiles of time of laceration repair for the infection group of patients were 290 min and 1,707 min, respectively. Time of laceration repair in patients who developed an infection had a bimodal distribution: an “early group” (< 750 min) and a “late group” (>1,000 min). Figure 4 shows time of laceration repair in those patients who developed wound infection. Patients were equally distributed in the infection and non-infection groups (five in each group). Of five patients in the “early group” who developed infection, three could be related to the presence of certain risk factors such as diabetes, obesity, crush injury and possible contamination. In the “late group” (greater than 1,000 min), risk factors were identified in one patient who had a recent history of pneumonia and malnutrition. Figure 5 shows a comparison between the infection group and non-infection group with relation to time. Figure 5 shows two combined histograms comparing the distribution of frequencies of infection and non-infection cases as the wound closure time for the lacerations increased. Neither of the histograms showed bell-shaped normal distribution. The histogram on the time of laceration in the non-infection group is skewed to the right where the mean time of laceration (578.90 min) was larger than the median (330 min). The histogram on the time of laceration in the infection group showed a peak at 300 min and a small bump at 1,700 min.

**Table 2:** Patients’ clinical measurements according to infection or non-infection in the study

**Measurement**	**Median (mean ±** **SD)**		
	**Infection (%)**	**Non-infection (%)**	***P*-values**
Length of laceration (cm)	3.5 (4.1 ± 3.1)	2.5 (2.7 ± 1.9)	0.17
Time of laceration (min)	867.0 (944.5 ± 3.1)	330.0 (578.9 ± 749.7)	0.03

*Wilcoxon-Mann-Whitney test.

There was no statistically significant difference for gender, patient age, wound location, wound length, mechanism, of injury, type of anesthesia used, suture material used and the presence of co-morbidities when comparing the patients in the infection group with the patients in the non-infection group.

## Discussion

Lacerations are one of the most common problems encountered in the Emergency Department and account for approximately 11 million visits annually [5]. Time from injury is an important factor in determining if a laceration should be closed primarily. However, controversy exists over when or if to close wounds that are delayed in presentation to the ED. It has been thought that “old” lacerations should be considered contaminated because of high levels of bacteria found in these wounds 6 to 8 h after laceration occurrence [6]. According to an older report, the “golden period” for the treatment of an acute wound has been defined as 6 h. This is based on laboratory and clinical studies on the doubling time of bacterial colonization progressing to an invasive infection, and from clinical outcomes describing the decreased risk for infection after cleaning and repairing the wound within that time [7,8]. Some researchers believe that lacerations that have not been grossly contaminated can be repaired without a significant increase in wound infection up to 12 h after the injury [9].

A published report by Lammers and colleagues stated that, in general, wounds older than 10 h were at a higher risk for infection, and hand lacerations older than 8 h had an increased risk of infection [10]. The current American College of Emergency Physicians policy on penetrating extremity wound management recommends that primary closure be completed no more than 8 to 12 h from the time of injury. It is generally believed that low risk lacerations such as those on the face, scalp and trunk with minimal contaminationcan be safely repaired up to 12 h after the time of injury. Moderate risk lacerations such as those on the extremities with poor vascular supply can be closed primarily after thorough irrigation within a 6- to 10-h period [11]. While the literature has suggested the use of clinical judgment in making a decision for laceration repair, there is no reported absolute time interval after which laceration repair should not be performed. It is stated that the time interval acceptable for laceration repair depends on each individual situation [12]. In the evaluation of a laceration, emergency physicians usually consider many characteristics of the wound to determine whether or not primary closure can be safely performed. These factors include time to laceration repair, location of the wound, the degree of contamination and the patient's predisposing medical conditions [13]. In another study, laceration characteristics associated with higher infection rates included jagged wound edges, stellate shape, injury deeper than the subcutaneous tissue, presence of a foreign body and visible contaminants [14]. A recent report has suggested that a time limit of 6 h for laceration repair may not be reasonable [15].

We included only simple and uncomplicated lacerations in this study, excluding all complicated lacerations. Since it is also generally accepted that lacerations involving sterile sites such as tendons, joints or bones are at increased risk for infection, we excluded these infection-prone lacerations. In addition, bites and puncture wounds are also infection prone and were therefore excluded. Presence of a foreign body was also considered a risk factor for infection [16], so lacerations with suspicion of a foreign body were excluded.

Generally, most lacerations were repaired by primary closure. It is believed there is a direct relation between the time from injury to closure of the laceration and the risk of subsequent infection, but the length of this “golden period” is highly variable. Thus, the time during which wound closure can safely be performed needs to be individualized based on the mechanism, site of injury and other host factors [17].

Our study using the aseptic technique in the repair of simple or uncomplicated lacerations indicates that the wound infection rate for repair of lacerations was increased at a wound closure time of 867 min as compared to 330 min. The difference in the median wound closure time for the lacerations in the infection group compared to those of the non-infection group was statistically significant (*p* = 0.03). The optimal time period for repair of simple lacerations may be longer than generally believed provided that the mechanism is not a crush injury and that the patient has no comorbid risk factors.

This study also reveals the unique and perhaps new finding that there is a bimodal time distribution in infection rates following laceration repair (Figure 4). Of five patients in the early infection group, three had some distinguishing factors such as co-morbidities, contamination or mechanism of injury. However, one patient in the “late group” had presence of a co-morbid condition (Table 3). It is possible that lacerations that are contaminated, have a crush mechanism or have preexisting comorbidities have devitalized tissue or have a heightened inflammatory state, such as diabetes, predisposing the wound to bacterial growth. This allows infections to occur more rapidly. However, lacerations that develop late infections do not have these risk factors. We believe that the presence of certain conditions such as diabetes, obesity or pneumonia may create a heightened inflammatory state and may result in the development of infection after laceration repair. It is, therefore, possible that the development of infection in this group may be time independent. We did not find a clear or absolute cutoff point after which the rate of laceration significantly rises. However, we did find a tendency for a small increase in infection rate after 1,000 min. This statement is also supported by the fact that most of the patients in the “early group” could be explained by the presence of risk factors compared to the late group.

**Table 3:** Characteristics of patients who developed infection after laceration repair

**Age**	**Site**	**Mechanism**	**Possible**	**Comorbidity**	**Time**	**Length**
**(years)**			**contamination**		**(min)**	**(cm)**
22	Extremity	Sharp	No	Obesity	220	4
50	Face	Crush	No	Diabetes,	287	1.5
				obesity		
27	Hand	Sharp	No	None	290	9
33	Hand	Sharp	No	None	300	1
40	Hand	Sharp	Yes	None	657	2.5
46	Extremity	Sharp	No	Pneumonia,	1,077	10
				malnutrition		
25	Hand	Sharp	No	None	1,439	1.5
26	Hand	Sharp	No	None	1,707	3.5
42	Extremity	Sharp	No	None	1,728	5
35	Extremity	Sharp	No	None	1,740	3.5

### Limitations

There are some limitations to our study. Although a written protocol for laceration management existed at our study site, practice variations among ED physicians were still possible. Because many ED physicians do believe in a "golden period" for closure of lacerations, the "more delayed lacerations" may not have been repairedbecause of the physician's belief that he or she would be violating the standard of care. We did not control for these practice variations among physicians.

In addition, our patient population that participated in this study may not be representative of all other ED patients with lacerations. This study is also limited by its small sample size, and can be considered an initial preliminary study aimed at uncovering a link between wound infection and time of laceration repair. If results support such a link, larger studies in the future may be conducted to validate our results. This relatively small sample size may have limited the statistical significance of this study. In addition, other possible factors, such as gender, wound location and mechanism, type of anesthesia and sutures, did not achieve statistical significance. Since development of infection was a rare event in our sample, lack of significance could be related to low power.

We have noted that there is a bimodal time distribution in infection rates ( 4). The study did not have enough power to determine unique characteristics in patients who have early versus late infections. However, we hypothesized that it may be related to the comorbid risk factors and mechanism of injury. Further studies with enough power should be designed to attempt to describe this bimodal time distribution more fully and to stratify on the above variables.

### Implications

The results of our study have demonstrated that there is an increase in the rate of infections for wound closure times after 1,000 min. Our study also suggests that there is a bimodal distribution between wound infections and wound closure times. Further studies with "greater power" are needed toclarify these points.We believe the results of this study will provide assistance in developing recommendations for when simple and uncomplicated lacerations may be primarily repaired without significantly increasing the risk of infection. Although our study did not demonstrate the importance of other factors in the development of wound infections as some other studies have, this may be due to the "low power" of our study in this regard. We are hopeful that future larger studies will demonstrate the importance of these factors and how they interact with wound closure time.

## Conclusion

In our study, development of infection after laceration repair was a rare event. The median wound closure time for the lacerations in the wound infection group was statistically significantly longer than in the non-infection group. Further large scale prospective studies are needed to confirm this relationship between wound infection and time of repair.

## Competing interest

The authors declare that they have no competing interests.

## Authors’ contributions

Data Collection & data entry (VL and RP). Protocol, development and manuscript writing (R. Data analysis and manuscript writing (ML). Statistical Support and data analysis (XS). All authors read and approved the final manuscript.
